# Supplementary material for: Curcumin Alleviates Aflatoxin B1-Induced Liver Pyroptosis and Fibrosis by Regulating the JAK2/NLRP3 Signaling Pathway in Ducks
Source: Foods. 2023 Feb 27;12(5):1006. doi: 10.3390/foods12051006 (PMC10000391; doi:10.3390/foods12051006)
Supplement: Supplementary file 1 [file foods-12-01006-s001.zip › foods-2145360-supplementary material.pdf]

Table S1

Primer sequences and amplification lengths of destination fragments.

Genes	Gene numbers	Primer sequences	Primer lengths (bp)	Product lengths (bp)
JAK2	XM_027447150.2	F:5' ACCTATTTGCACAGTGGCGAGATG 3′	24	139
		R:5' AGTGGTGTTTGGTCCCTTTCTTTGG 3′	25	
STAT3	XM_038168717.1	F:5' CGGCGGAGTTCAAGCACCTG 3′	20	85
		R:5' GTCACGATCAGCGAGGCATCAC 3′	22	
NLRP3	XM_005029958.4	F:5' CCAGCCTGAAGATCGGAGACCT 3′	22	143
		R:5' AGGAGCCACCCTAGAGGAGAGT 3′	22	
ASC	XM_013201308.1	F:5' CAGCATTCTGGATCGGCTCT 3′	20	90
		R:5' ATTTTCTCCTGCCTGATGCTT 3'	21	
Caspase-1	XM_027446016.1	F:5' TCGGTGCTGGTGTCTGACTCA 3′	21	151
		R:5' AGACGGTATCAGGTGTGGAGGA 3′	22	
IL-1β	DQ393268.1	F:5' CAAGCTCTACATGTCGTG 3′	18	119
		R:5' CAGGCGGTAGAAGATGAAG 3′	19	
IL-18	XM_027444356.1	F:5' CTGATGACGATGAGCTGGAA 3′	20	120
		R:5' CAAAAGCTGCCATGTTCAGA 3′	20	
α-SMA	XM_005029417.5	F:5' ATTCTTGCCTCCCTCTCTACCTTCC 3′	25	88
		R:5' ACTTGCGGTGAACAATGGATGGG 3′	23	
Col-I	XM_038173913.1	F:5' GGTGGTGGATATGAAGTTGGCTACG 3′	25	130
		R:5' GGGTCAGCAGGGTCTCAATTTGG 3′	23	
TGF-β	XM_027454349.2	F: 5' GGATGGAGCGACTTCAACGAGAAC 3′	24	103
		R:5' AGCCGCCTTCGTGTCAGATTATTC 3′	24	
β-actin	EF667345.1	F:5' ATGTCGCCCTGGATTTCG 3′	18	165
		R:5' CACAGGACTCCATACCCAAGAAT 3′	23	

Table S2

The sources of the antibodies.

Antibodies	Company	Catalog No.	Dilution ratio
JAK2	Beyotime, China	AF1489	1:1000
p-JAK2	Beyotime, China	AF1486	1:1000
STAT3	Beyotime, China	AG3322	1:1000
p-STAT3	Beyotime, China	AF5944	1:1000
NLRP3	Wanleibio, China	WL02635	1:2000
ASC	Wanleibio, China	WL02462	1:500
Caspase-1	Wanleibio, China	WL02996	1:1000
GSDMD	Cell Signaling Technology, USA	69469	1:1000
α-SMA	Beyotime, China	AF1507	1:1000
Col-I	Beyotime, China	AF1840	1:1000
TGF-β	Beyotime, China	AF0297	1:1000
GAPDH	Beyotime, China	AF2819	1:2000
Mouse IgGκ BP-HRP	Santa Cruz, USA	sc-516102	1:5000
HRP Goat Anti-Rabbit IgG	ABclonal, China	AS014	1:5000
